# Yellow fever in the diagnostics laboratory

**DOI:** 10.1038/s41426-018-0128-8

**Published:** 2018-07-12

**Authors:** Cristina Domingo, Rémi N. Charrel, Jonas Schmidt-Chanasit, Hervé Zeller, Chantal Reusken

**Affiliations:** 1 Highly Pathogenic Viruses (ZBS 1), Centre for Biological Threats and Special Pathogens, Robert Koch Institute, WHO Collaborating Centre for Emerging Infections and Biological Threats, 13353 Berlin, Germany; 2 UMR “Unité des Virus Emergents” (UVE: Aix-Marseille Univ – IRD 190 – Inserm 1207 – IHU Méditerranée Infection), Marseille, France; 3 Faculte de Medecine de Marseille, 13005, Marseille, cedex 05 France; 4 Bernhard Nocht Institute for Tropical Medicine, WHO Collaborating Centre for Arbovirus and Haemorrhagic Fever Reference and Research, 20359 Hamburg, Germany; 5 European Centre for Disease Prevention and Control (ECDC), 171 65 Solna, Sweden; 6 Department of Viroscience, WHO Collaborating Centre for Arbovirus and Haemorrhagic Fever Reference and Research, Erasmus MC, 3000 CA Rotterdam, The Netherlands; 7 Present Address: Institut Pasteur, Direction of International Affairs, 75015 Paris, France

## Abstract

Yellow fever (YF) remains a public health issue in endemic areas despite the availability of a safe and effective vaccine. In 2015–2016, urban outbreaks of YF were declared in Angola and the Democratic Republic of Congo, and a sylvatic outbreak has been ongoing in Brazil since December 2016. Of great concern is the risk of urban transmission cycles taking hold in Brazil and the possible spread to countries with susceptible populations and competent vectors. Vaccination remains the cornerstone of an outbreak response, but a low vaccine stockpile has forced a sparing-dose strategy, which has thus far been implemented in affected African countries and now in Brazil. Accurate laboratory confirmation of cases is critical for efficient outbreak control. A dearth of validated commercial assays for YF, however, and the shortcomings of serological methods make it challenging to implement YF diagnostics outside of reference laboratories. We examine the advantages and drawbacks of existing assays to identify the barriers to timely and efficient laboratory diagnosis. We stress the need to develop new diagnostic tools to meet current challenges in the fight against YF.

## Introduction

The last two years have seen a re-emergence of yellow fever (YF) in countries in Africa and the Americas, which brings into acute focus the need for effective tools and protocols in medical practice and public health policy against this arboviral disease. Suitable YF diagnostics in humans, non-human primates (NHPs) and vectors constitute first-line defenses because timely laboratory confirmation of suspected YF cases is essential for effective outbreak control and the prevention of further spread. Meeting the current and future challenges of YF epidemics will require building up laboratory preparedness and proficiency, especially in the geographic areas of disease endemicity, and this build up should be informed by a thorough understanding of yellow fever virus (YFV) diagnostics. Here, we survey the field of YFV laboratory methodology in the context of the YF epidemiological situation in early 2018 as experts associated with the European Centre for Disease Prevention and Control (ECDC) Emerging Viral Diseases-Expert Laboratory Network (EVD-LabNet). We hope that this review of the strengths and limitations of the YF diagnostic toolkit, along with the included background information on the pathogen and disease, will assist diagnostics laboratories and public health officials in targeting areas of their practice for upgrade and research in the context of the ongoing fight against YF epidemics.

## The YF epidemiological landscape, 2015–2018

Urban outbreaks of YF were declared in Angola in December 2015 and soon after in the Democratic Republic of the Congo (DRC). WHO declared the end of these outbreaks in January 2017 with a final register of 7334 suspected cases, 965 of which were laboratory-confirmed cases, including 137 fatalities^1^. In 2016, an unrelated outbreak was declared in Uganda^2^ and sporadic YF cases were also detected in Chad, Ghana, the Republic of Congo, and Guinea^3^. Nigeria is currently dealing with an active YFV outbreak^4^.

The cornerstone of the WHO coordinated international response to stop the transmission and anticipated spread of YF to other countries consisted of reactive and pre-emptive mass vaccination campaigns launched in Angola and the DRC^5^. A shortage of emergency vaccine supplies, however, led to a dose-sparing strategy implemented during the latest vaccination campaigns in Africa, which used one-fifth of the original dose^6^. Preliminary estimates of the seroconversion rate are not divergent from those achieved by full-dose vaccination^7–9^, but data are scarce on the duration of the immunity imparted by this approach.

In December 2016, a YF outbreak was declared in Brazil with over 3240 suspected (779 confirmed) human cases as of 13 December 2017^10^. The number of cases declined from May 2017 onwards, but from July 2017 to 13 March 2018, 920 human cases (300 deaths) were reported in the states of Minas Gerais, São Paulo, Rio de Janeiro, and Espírito Santo and in the Federal District^11^. An alarming number of epizootics in NHPs have been reported from different Brazilian states during the considered time period, with the Sao Paulo metropolitan area accounting for 40% of them^12^. The presence of epizootics and confirmed cases near the urban areas of São Paulo and Rio de Janeiro and in municipalities that were previously considered not at risk of YF is a worrying trend because much of the population in these areas remain unvaccinated^12^. This outbreak, the most severe for several decades in Brazil, raises the concern that YF infections are no longer confined to jungle and remote areas as sylvatic transmission is now also occurring in the periphery of densely populated cities^13^.

In October 2017, the Brazilian public health authorities responded to the recorded epizootics with vaccination campaigns in the Northern areas of the city of São Paulo in an effort to prevent human cases in areas bordering epizootic prevalence and to control the risk of an urban outbreak^14^. A massive vaccination campaign took place in São Paulo in early 2018 using a fractionated dose of the vaccine^15^. Due to a limited vaccine stock, the Brazilian Ministry of Health adopted the WHO recommendation to administer a single dose of YF vaccine^6^; however, this strategy has generated some controversy regarding the duration of immunity^16,17^, and this decision is considered an emergency response to be re-evaluated in the short term^18^.

Colombia, Peru, Bolivia, Suriname, Ecuador, and French Guiana^11^ have also reported cases of epizootic and sylvatic YF in recent years. An aggravated risk of further disease spread was suggested by the increased incidence of sylvatic YF and the detection of human cases in Peru^11^. Likewise, Bolivia reported in February 2017 the first YF case in decades, which involved a non-vaccinated tourist, and four additional cases were confirmed in this country from May to July 2017^19^.

Further international spread to areas with susceptible populations and competent mosquito vectors is a grave concern^20^. WHO considers the risk of YF spread at the regional level in the Americas to be low given the high vaccination coverage in Brazil’s neighboring countries, but the detection in August 2017 of a human case in French Guiana near the border with Brazil shows that the risk is real. At a global level, the risk remains restricted to non-vaccinated travelers^12^. Recently, the Evandro Chagas Institute reported the detection of YF in *Ae*. *albopictus* mosquitoes in 2017^12^. The European Centre for Disease Prevention and Control (ECDC) stated a risk of uncertain magnitude for regions harboring endemic *Ae*. *albopictus* populations^20,21^. In non-endemic regions, such as Europe, preparedness and capability assessment activities for reference laboratories have to be undertaken to guarantee a timely diagnosis of suspected cases in travelers returning from areas with increased YFV circulation^22^.

This landscape of YF outbreaks has prompted WHO and partner organizations (UNICEF and GAVI) to revise their long-term YF strategy for the next 10-year period (2017–2026). The novel EYE (Eliminating Yellow fever Epidemics) strategy is a global and comprehensive long-term (2017–2026) scheme that builds on lessons learnt from recent outbreaks and aims to protect at-risk populations, prevent the international spread of the disease, and readily contain outbreaks^23^.

## The pathogen

**Fig. 1:**
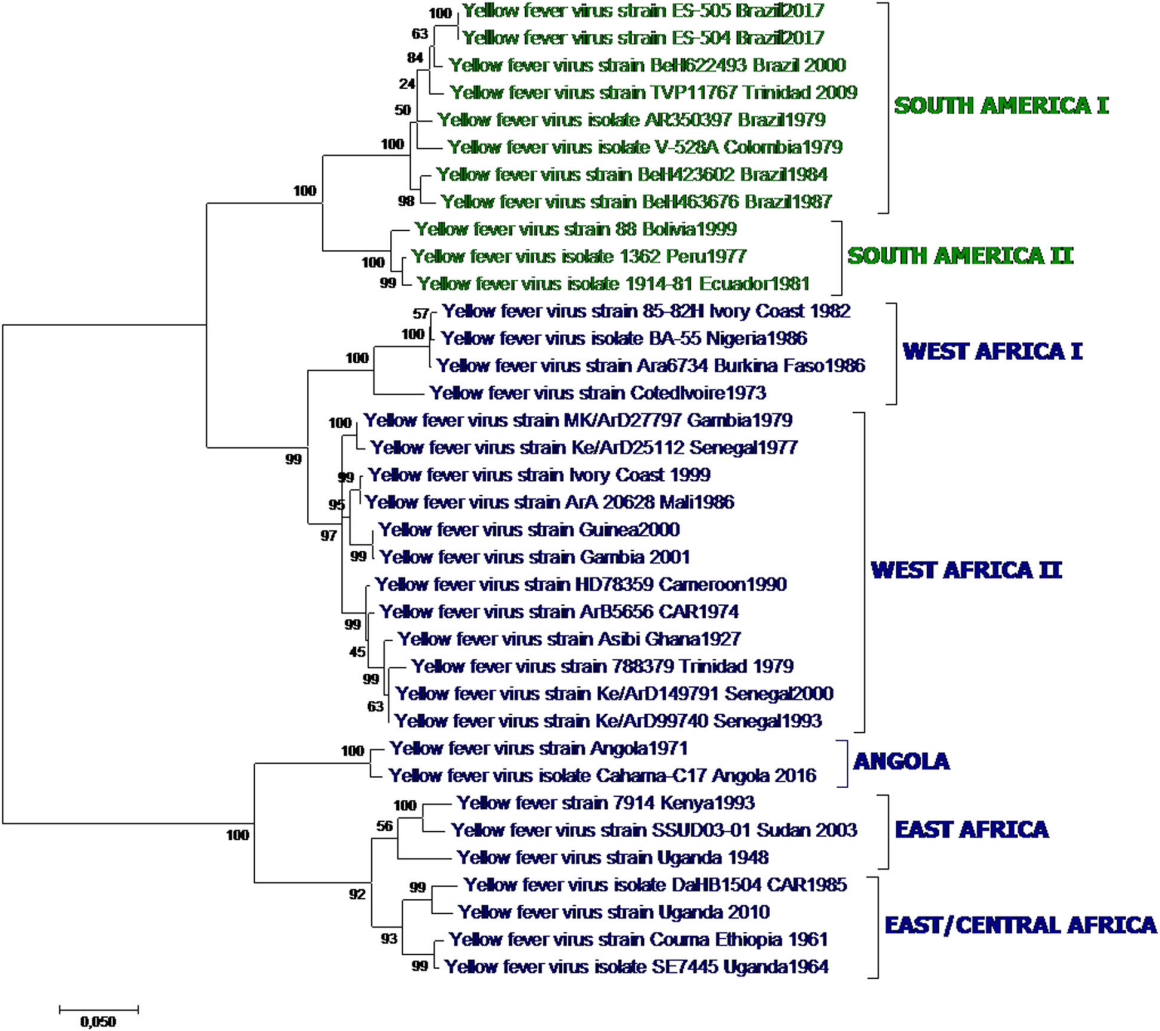
Yellow fever phylogenetic analysis showing major YFV genotypes, based on alignment of a 1428 nt region of the prM-E junction region for 36 representative African and American YFV strains (Table 1) using the Maximum Likelihood method based on the general time reversible model (GTR). Individual strains are defined by name and country/year of isolation. Bootstrap values (500 replicates) for major branches are indicated

YFV is an enveloped virus with a single-stranded RNA genome and is a member of the genus *Flavivirus*, family *Flaviviridae*. Other flaviviruses of major importance to human health are dengue virus (DENV), West Nile virus (WNV), Zika virus (ZIKV), Japanese encephalitis virus (JEV), and tick-borne encephalitis virus (TBEV). YFV belongs to the YFV serogroup of mosquito-borne flaviviruses and is transmitted by *Aedes* mosquitoes. The circulating YFV strains constitute a single serotype, but seven major genotypes have been described (Fig. 1), five of which circulate in Africa and two in South America^24^.

The evolutionary rates described for YFV are similar across the various genotypes and are estimated to be lower than those of other mosquito-borne flaviviruses, such as DENV^25^. Estimated genetic variance within the clade is 10–23% at the nucleotide level for the five African genotypes and 5% for the two American genotypes. The African genotypes are up to 16% dissimilar from the American genotypes^26^.

The strain of the recent Angola and DRC outbreaks is most closely related to that responsible for the 1971 Angola outbreak;^27^ likewise, preliminary sequencing of the 2016 Uganda strain showed that it is most closely related to a strain isolated in this country in 2010^28^, which was of East/Central Africa genotype. Whole-genome sequence analysis of the current Brazil outbreak strain assigned it to South American genotype 1E^29^, which arose in Brazil during the 2008–2009 epidemics^30^, and identified eight mutations of possible functional importance that are still under investigation^31^. These changes, however, are not expected to affect the efficacy of currently available vaccines^31^.

## Epidemiology and geographical spread

YFV is endemic and intermittently epidemic to tropical and subtropical areas of South America and Africa. Africa accounts for 90% of the global burden of YFV. The true incidence of YF is unknown because of insufficient reporting, ground surveillance, and limited access to specific diagnostics for YF and other common pathogens in differential diagnosis (i.e., malaria and viral hepatitis). Estimates based on African data sources from 2013 put the incidence at 84,000–170,000 severe cases per year, causing 29,000–60,000 deaths^2^. Autochthonous YFV transmission has not been detected in Asia yet, despite a large susceptible population and widespread competent mosquito vectors.

YFV is maintained in a sylvatic transmission cycle between NHPs and jungle mosquitoes (*Aedes spp*. in Africa*, Haemagogus spp*. and *Sabethes spp*. in South America), with humans getting infected when they enter forested areas for occupational, tourism, or leisure activities. The arrival of viremic persons in a densely populated urban environment could initiate a transmission cycle between humans and competent vectors present in the area, mainly *Ae. aegypti*. This so-called urban transmission cycle would have devastating consequences in Brazil, similar to those in the recent Angola and DRC outbreaks. Thus far, *Ae. aegypti* has not been involved in the ongoing Brazilian outbreak^11,12^, and the recent detection of YF in *Ae. albopictus* in rural areas in the state of Minas Gerais in Brazil deserves further investigation^12^. In Africa, the savannah transmission cycle connects the sylvatic and urban cycles by involving *Aedes* mosquitoes (*Ae. furcifer*, *Ae. vittatus*, *Ae. luteocephalus* and *Ae. africanus* in West Africa; *Ae. africanus* and *Ae. simpsoni* in East Africa) that feed on both humans and monkeys^32^.

NHPs involved in the sylvatic transmission of YFV in the Americas belong to the genera *Aotus*, *Alouatta*, *Cebus*, *Ateles*, *Callithrix*, and *Saimiri*. American NHPs exhibit different susceptibility to YFV. *Alouatta* (howler) monkeys are particularly susceptible, and they frequently die after YFV infection due to liver and renal failure and hemorrhage caused by the infection, whereas *Callithrix* and *Cebus* monkeys exhibit different grades of resistance. On the other hand, African NHPs experience inapparent infections while viremic^33^. The use of NHPs as sentinels for the early detection of the circulation of YFV is a proven useful surveillance tool to evidence the presence of sylvatic activity of the virus, leading to the activation of countermeasures (i.e., vector control and population vaccination) to control the spread of the virus and the occurrence of epidemics^34,35^. The collection of appropriate material for diagnosis is an essential part of epizootics investigation, and proper storage and transport are key to the reliability of the laboratory results. The prioritized samples for epizootics investigation are blood, serum, and tissues (liver, spleen, kidneys, heart, lung and brain, when possible). In the laboratory, viral isolation, genome detection, serology, histopathology, and immunohistochemistry (IHC) exams are attempted^36^.

During the recent Brazilian outbreak, a number of YFV-infected marmosets were detected in urban areas. Given the habitat versatility of marmosets, whose range includes forest edge areas, the question has been raised about their role not only as sentinels but also as a link in the transmission cycle of the virus and the spread of YF to urban areas^37^.

Except for one case of nosocomial transmission in the 1930s^38^, there are no reports of direct human-to-human YFV transmission outside the laboratory (see Biosafety below). However, transplacental-^39,40^, breastfeeding-^41–43^, and blood donation-based viral transmission^44^ has been described for the live attenuated YFV vaccines.

Lastly, the recent discovery of sexual transmission of Ebola virus (EBOV)^45^ has prompted investigations of this alternative, previously overlooked mode of transmission. Sexual transmission of ZIKV has been demonstrated^46^. Clinical and experimental investigations of YFV via sexual transmission are thus warranted.

## YF disease

As in other flavivirus infections, most YFV-infected people are asymptomatic. When present, symptoms may include fever, headache, nausea, muscle pain, backache, vomiting, jaundice, and bleeding from the mouth, nose, eyes or stomach. In 25–50% of cases, the disease can progress into full hemorrhagic syndrome with multiorgan failure^47^. Treatment for YF is only supportive. The clinical course comprises three stages: infection, remission, and intoxication, often without clear stage demarcation. During the so-called period of remission, starting 3–4 days after the onset of symptoms, clinical signs subside and the patient may either go into remission or conversely deteriorate into the intoxication phase, which is characterized by high fever, nausea, vomiting, abdominal pain, and changes in consciousness^48^. Jaundice from excess bilirubin arises from liver cell damage (uniquely among hemorrhagic fevers), along with epistaxis, bleeding of the oral mucosa, hematemesis, and petechial hemorrhage. The patients may further deteriorate rapidly, and 20–50% will die 7–10 days after the onset of symptoms. Jaundice and increased liver enzymes, specifically serum aspartate aminotransferase (AST) levels over 1200 UI/l, have been correlated to disease severity and higher mortality^49^. Renal failure is also a manifestation of severe and fatal YF, and blood urea nitrogen (BUN) levels over 100 mg/mL were associated with an elevated risk of death^49^.

Vaccination against YF has been associated with rare cases of viscerotropic (yellow-fever vaccine-associated viscerotropic disease, YEL-AVD) and neurotropic disease (yellow-fever vaccine-associated neurotropic disease, YEL-AND)^50^. YEL-AVD clinical presentation is similar to wild-type YF disease with nonspecific initial symptoms, including fever, headache, malaise, myalgia, arthralgia, nausea, vomiting, and diarrhea, starting 2–8 days after vaccination. Jaundice can appear, along with thrombocytopenia and the elevation of hepatic transaminases, total bilirubin, and creatinine. Severe YEL-AVD is characterized by hypotension, hemorrhage, and acute renal and respiratory failure, leading to multiorgan system failure. Similarly, YEL-AND includes post-vaccinal encephalitis but also autoimmune disease with central or peripheral nervous system involvement, such as acute disseminated encephalomyelitis or Guillain-Barré syndrome. The clinical presentation varies but includes high fever and headache associated with confusion, lethargy, encephalitis, encephalopathy, and meningitis^51^.

## Infection kinetics

Awareness of YFV infection kinetics is essential in designing optimal sampling strategies because the timing of sample-taking and the nature of the biological sample constrain diagnostic interpretation.

### Viraemia

YFV infection by a mosquito bite typically has a 3–6-day incubation period (range: 2–9 days)^52^. It was traditionally assumed that YFV could be detected afterwards in the serum, plasma, or whole blood of symptomatic patients during the first 5 days of illness. Molecular diagnostics have now shown that viral RNA can be efficiently detected for longer periods in the blood and autopsy tissues of severe cases^53–59^ and up to 20 days after the onset of symptoms^52,60,61^.

Data are scarce on the detection of YFV in other body fluids, such as urine, saliva, or semen, following natural infection. It has been demonstrated that YFV can be detected for a longer time period in urine than in serum, and can be detected up to 25 days post-inoculation in cases of suspected adverse events after YF vaccination^62^. Recently, YFV RNA has been efficiently detected in urine samples from natural infection cases^60,61,63^ and in semen up to 20 days after disease onset^61^, which is also a substantially longer detection time than in serum. These observations strongly suggest urine as a valuable diagnostic sample for YF as observed previously for other flaviviruses, such as WNV and ZIKV, which deserves more attention. Likewise, the transmission of YF vaccine virus to babies born to vaccinated mothers suggests the presence of YFV in breast milk^41–43^.

### Humoral immune response

Typically, anti-YFV IgM antibodies develop within a few days after the onset of illness with flaviviruses and can generally be detected for up to 3 months, whereas IgG antibodies develop within days subsequent to the IgM response and can be detected for years afterwards. The persistence of IgM antibodies for longer periods has been reported in a small percentage of vaccinees, which could interfere with diagnostic testing^64^. IgM production in response to secondary flavivirus infection (e.g., in YF cases with a prior history of infection by DENV) may be absent or small, hampering the serological identification of acute cases^33,64^.

## YF diagnostics: state of the art

The clinical diagnosis of YF is problematic because the symptoms resemble those of a wide range of diseases, including dengue, other hemorrhagic viral diseases, leptospirosis, viral hepatitis, and malaria. All of these diseases have to be considered in differential diagnosis, and laboratory confirmation is essential. Detection of YFV-specific IgM in the absence of recent YF vaccination and negative diagnosis (including IgM antibodies) for other flaviviruses is considered confirmatory of YF. More robust corroboration of YFV infection, however, is provided by immunohistochemical detection of the YFV antigens, PCR amplification of YFV genomic sequences from blood or solid tissues, or by a test for viraemia involving the cultivation of YFV infectious particles. Generally, these assays are performed only in a few national or international reference laboratories.

### YFV molecular diagnostics

Eleven quantitative real-time RT-PCR assays for molecular detection of the YFV genome have been described as of March 2018^65–75^. In addition, four alternative assays oriented to field and point-of-care diagnosis have been reported in recent years based on isothermal amplification of the viral genome^69,76–78^.

**Fig. 2:**
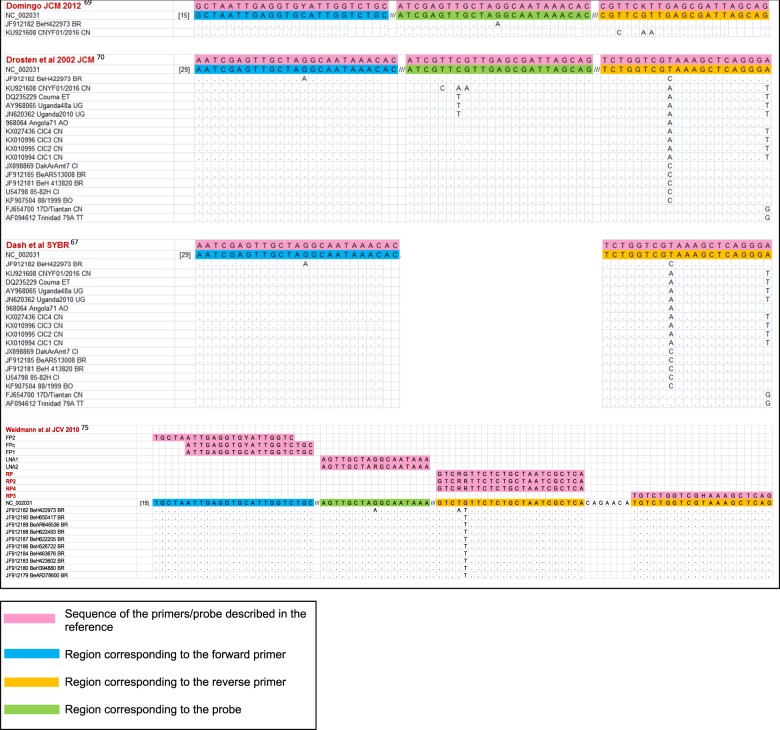
Alignment of the primers and probes of shortlisted assays against relevant YFV target sequences. The figure is restricted to the four assays described in references^67,69,70,75^, which generated the fewest mismatches overall in the comparison with the reference set of 61 YFV genomic sequences (Table 1). Perfectly matched YFV sequences are not shown. Primer and probe sequences are written 5′ to 3′ except for the reverse primers at the right edge of the figure, which are represented by the reverse-complement of the oligonucleotide sequence

**Fig. 3:**
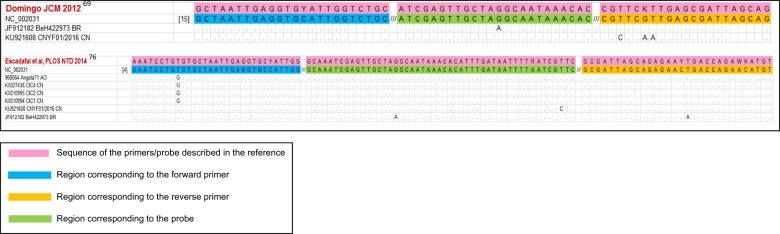
Alignment of the primers and probes of shortlisted assays against relevant YFV target sequences. The figure is restricted to the two assays described in references^69,76^, which generated the fewest mismatches overall in the comparison with the reference set of 61 YFV genomic sequences (Table 1). Perfectly matched YFV sequences are not shown. Primer and probe sequences are written 5′ to 3′ except for the reverse primers at the right edge of the figure, which are represented by the reverse-complement of the oligonucleotide sequence

In selecting an assay for the diagnosis of natural YFV infections, one should avoid those designed specifically for vaccine strains^65,71^ because their detection of wild strains would be less reliable^79^. In this work, we have reviewed assay specificity in the context of the differences between American and African strains and sequence diversity among the strains currently circulating in endemic areas. We matched the target sequences of published RT-PCR and isothermal amplification assays against an alignment of 61 complete YFV genomic sequences from GenBank (Table 1) to check the capability of each assay to detect all strains. Among the 14 assays described in international journals^65–78^, most were clearly unsuitable for many strains owing to excessive mismatches between the primers and/or probe and the target genome. Four real-time RT-PCR assays, namely, two TaqMan^69,70^, one LNA^75^ and one SYBR Green-based assay^67^, were studied in detail and are predicted to detect all considered strains (Fig. 2). Similar in silico analyses of published isothermal amplification protocols predicted functional sets of primers and probes in two assays (Fig. 3), whereas the number of mismatches in the other two assays^77,78^ made them unsuitable for at least some of the wild strains. The generic qRT-PCR protocol described in ref. ^69^ is currently recommended by PAHO as the benchmark method for YF diagnosis in reference laboratories. This robust assay performs equally well with a variety of commercial reagents, has been extensively validated and implemented in several laboratories, and provides a profile of sensitivity and specificity appropriate for reliable case detection^69^. Used as the assay of reference, it has enabled the homogenization and standardization of laboratory data between different settings in the current Brazilian outbreak.

**Table 1:** Yellow fever virus strains (A: vaccine strains, B: wild type strains) included in the evaluation of suitability of published molecular detection assays

A Yellow fever vaccine strains	
**GenBank accession**	**Name**
U21055.1	YFV French neurotropic strain
U21056.1	YFV French viscerotropic strain
X03700.1	YFV 17D vaccine strain
U17067.1	YFV vaccine strain 17D-213
U17066.1	YFV vaccine strain 17DD
DQ100292.1	YFV strain 17DD-Brazil
DQ118157.1	YFV isolate YF-AVD2791-93F/04
JN628281.1	YFV strain 17D Flavimun TVX
JN628280.1	YFV strain 17D Flavimun WSL
JN628279.1	YFV strain 17D RKI
JN811143.1	YFV 17D YF-VAX Series C P11
JN811142.1	YFV 17D YF-VAX Series B P11
JN811141.1	YFV 17D YF-VAX Series A P11
JN811140.1	YFV 17D YF-VAX Series A P1
KF769015.1	YFV strain 17D-204
GQ379163.1	YFV strain case #2
GQ379162.1	YFV strain case #1
JX503529.1	YFV strain YF/Vaccine/USA/Sanofi-Pasteur-17D-204/UF795AA/YFVax
FJ654700.1	YFV 17D/Tiantan
NC_002031.1	YFV, NCBI reference sequence

B Yellow fever wild type strains			
GenBank accession	Sequence name	Country/year	Genotype
KU921608.1	YFV isolate CNYF01/2016	China ex Angola/2016	Angola
AY968064.1	YFV strain Angola71	Angola/1971	Angola
KX027336.1	YFV isolate CIC4	China ex Angola/2016	Angola
KX010996.1	YFV isolate CIC3	China ex Angola/2016	Angola
KX010995.1	YFV isolate CIC2	China ex Angola/2016	Angola
KX010994.1	YFV isolate CIC1	China ex Angola/2016	Angola
KF907504.1	YFV strain 88/1999	Bolivia/1999	Angola
AY968065.1	YFV strain Uganda48a	Uganda/1948	East Africa
DQ235229.1	YFV strain Couma	Ethiopia/1961	East/ Central Africa
JN620362.1	YFV strain Uganda 2010	Uganda/2010	East/ Central Africa
JF912190.1	YFV strain BeH655417	Brazil/2002	South America I
JF912189.1	YFV strain BeAR646536	Brazil/2001	South America I
JF912188.1	YFV strain BeH622493	Brazil/2000	South America I
JF912187.1	YFV strain BeH622205	Brazil/2000	South America I
JF912186.1	YFV strain BeH526722	Brazil/1994	South America I
JF912185.1	YFV strain BeAR513008	Brazil71992	South America I
JF912184.1	YFV strain BeH463676	Brazil/1987	South America I
JF912183.1	YFV strain BeH423602	Brazil /1984	South America I
JF912182.1	YFV strain BeH422973	Brazil /1984	South America I
JF912181.1	YFV strain BeH413820	Brazil /1983	South America I
JF912180.1	YFV strain BeH394880	Brazil /1981	South America I
JF912179.1	YFV strain BeAR378600	Brazil/1980	South America I
KY885000	YFV strain ES-504	Brazil/2017	South America I
KY885001	YFV strain ES-505	Brazil/2017	South America I
JX898869.1	YFV isolate DakArAmt7	Cote d’Ivoire/1973	West Africa I
AY603338.1	YFV strain Ivory Coast 1999	Cote d’Ivoire/1999	West Africa I
U54798.1	YFV strain 85-82H Ivory Coast	Cote d’Ivoire/1982	West Africa I
AF094612.1	YFV strain 79A/788379	Trinidad/1979	West Africa II
KF769016.1	YFV strain Asibi	Ghana/1927	West Africa II
JX898881.1	YFV isolate ArD181439	Senegal/2005	West Africa II
JX898880.1	YFV isolate ArD181564	Senegal/2005	West Africa II
JX898879.1	YFV isolate ArD181676	Senegal/2005	West Africa II
JX898878.1	YFV isolate ArD181250	Senegal/2005	West Africa II
JX898877.1	YFV isolate ArD181464	Senegal/2005	West Africa II
JX898876.1	YFV isolate ArD156468	Senegal/2001	West Africa II
JX898875.1	YFV isolate ArD149815	Senegal/2000	West Africa II
JX898874.1	YFV isolate ArD149194	Senegal/2000	West Africa II
JX898873.1	YFV isolate ArD149214	Senegal/2000	West Africa II
JX898872.1	YFV isolate ArD114972	Senegal/1995	West Africa II
JX898871.1	YFV isolate ArD114896	Senegal/1995	West Africa II
JX898870.1	YFV isolate ArD121040	Senegal/1996	West Africa II
JX898868.1	YFV isolate HD117294	Senegal/1995	West Africa II
AY572535.1	YFV strain Gambia 2001	Gambia/2001	West Africa II

Two additional qRT-PCR methods have been recently reported for the detection and initial identification of YFV wild and vaccine strains by qRT-PCR as an alternative approach to sequencing. One method consists of a YFV American strain-specific qRT-PCR duplexed with a specific YF vaccine qRT-PCR^74^. The second approach consists of the use of a reference generic YF qRT-PCR method for case detection^69^, coupled to a generic YFV vaccine-strain qRT-PCR methodproviding a global approach covering all strains^80^. Since the specificity of these methods is based on single nucleotide differences between wild-type and vaccine strains, the identification of a natural infection versus a vaccine-related adverse event in a vaccinated patient in an area with no reported YF infections should be carefully characterized to exclude the possibility of mutations in the vaccine strain that could lead to case misclassification.

It was suggested recently that next-generation sequencing may be useful for the diagnosis of emerging infectious diseases in general, and hemorrhagic viral diseases specifically, as samples are analyzed in a non-biased manner with no assumptions regarding the pathogen involved. This approach may be valuable in identifying the causative agent at outbreak onset and in sporadic cases and characterizing novel pathogens or suspected new strains of known viruses, but its wider application for routine YF diagnostics has not been suggested^81,82^.

Commercial kits for YFV genome detection are provided by Genekam, Genesig, ViPrimePLUS, PCRmax, LifeRiver Bio-Tech, Altona, and Fast-Track Diagnostics (FTD). FTD combines a test for YFV with *Brucella spp*, *Streptococcus pneumonia* and *Coxiella burnetii* detection within a Tropical Fever Africa panel. The stated detection threshold is 100 copies/reaction for the ViPrimePLUS assay, and a more sensitive 1,000 copies/ml threshold for the FTD and LifeRiver assays; no information is provided for the other assays. The FTD Tropical Fever Africa and LifeRiver kits carry the conformity mark for European Economic Area regulations (CE). From Genekam, the dedicated YFV kit is CE-marked, but the three kits allowing YFV detection in combination with other pathogens (YFV + ZIKV + CHIKV; YFV + EBOV + Rift Valley fever virus; YFV + ZIKV) are not. Assays distributed by Genesig, VirPrimePLUS, Altona, and PCRmax are not CE-marked. To the best of our knowledge, no peer-reviewed reports on the evaluation of these kits using clinical samples from natural infections are available.

The recently sequenced YFV Brazilian strain displays eight non-silent base substitutions relative to previous isolates, seven in the NS3 and NS5 genes and one in the C protein gene^31^. Caution is advised about using any assays targeting these genes, but the new mutations should not affect the performance of the selected assays (Figs. 2 and 3), which all target the 5ʹ-noncoding region of the genome.

Special consideration is given to the use of paraffin-embedded or formalin-fixed samples for the molecular detection of YFV RNA. Even though qRT-PCR assays amplifying short regions of the YFV genome are very useful for these samples and support the results of histopathology or IHC, the detection of YFV RNA in these samples is, however, not entirely consistent, and false negatives might occur due to RNA degradation or damage during sample preparation and extraction, the generation of secondary structures during prolonged formalin fixation or the presence of inhibitors78.

### Serology: an overview of current knowledge and methodologies

#### Limitations of the serological diagnosis of YFV infections

The serology criteria for YFV infection are the detection of either YFV-specific IgM species or a four-fold or greater increase in anti-YFV IgG antibody titers in acute and convalescent samples^83^. YF serological diagnosis, however, is complicated by cross-reactivity with other members of the genus Flavivirus (such as DENV, WNV, Saint Louis encephalitis virus (SLEV), or ZIKV), the phenomenon known as original antigenic sin, and the lack of extensively validated commercial assays.

Prior immunity to DENV is the most frequent confounder generating non-specificity in current serological tests^84^, which is relevant as DENV and YFV share distribution areas in the Americas and Africa, and a DENV vaccine has recently been introduced into some countries in the Americas.

The approach to YFV serological diagnosis recommended by WHO differs slightly depending on the epidemiological context, i.e., outbreaks versus endemicity or non-endemicity areas. The guidelines take into account the presence of antigenically related viruses but also the high prevalence of vaccination in the areas affected and the usual concomitant implementation of YFV vaccination campaigns^85^. Correct attribution of severe symptoms to either natural infection or the adverse effects of vaccination is particularly difficult in outbreak contexts, where the antigenic similarity between wild-type and vaccine strains precludes unambiguous serological identification. The distinction is only currently possible through molecular characterization of the causative agent by sequence analysis or alternatively by the molecular methods mentioned previously.

#### Assays for serological diagnosis of YFV infections

A variety of in-house methods have been described for YF serodiagnosis, surveillance purposes, or confirmation of the immune response to vaccination. Serodiagnosis is often requested from reference laboratories for individuals in which special circumstances may compromise the response to vaccination, such as pregnancy, immunosuppressive treatment, HIV infection, or other instances of inborn or acquired immunodeficiency.

The plaque reduction neutralization (PRNT) assay, or virus neutralization test (VNT), is the most specific method for the detection of YFV antibodies and the current “gold standard” for flavivirus differential diagnosis. A degree of cross-reactivity with other flaviviruses (e.g., DENV and ZIKV) has been observed in the PRNT assay during secondary flavivirus infections^86^, with the higher stringency PRNT 90% assay providing greater specificity than other assays (although sensitivity may decrease as a trade-off). PRNT assays, however, require specific cell culture facilities, standardized controls and well-trained personnel for reproducible results. This limitation confines PRNT to reference laboratories, which may create a diagnosis bottleneck in outbreak situations. Moreover, the time to final interpretation of results, which is usually 4–7 days, delays diagnosis and is not best-suited to decision-making during an outbreak response, i.e., regarding vaccination deployment.

Hemagglutination inhibition (HI) and complement fixation (CF) methods have been used in the past for the serodiagnosis of YF, but they have been used less frequently in recent years as they are non-discriminant of the IgM/IgG antibody class and perform poorly in comparison to alternative assays^79,87^.

Other tests that are currently used for the detection of IgM and IgG antibodies against YFV include in-house indirect immunofluorescence methods (IIF), which require well-trained personnel for correct interpretation, and ELISA^79,87,88^, MAC-ELISA^89^, and ELISA inhibition tests^89,90^. More recently, a multiplex microsphere immunoassay (MIA) test has been described for the detection of arboviral antibodies, including those against YFV^91^.

The United States Centers for Disease Control and Prevention (US CDC) have traditionally provided (via WHO) testing reagents for a MAC-ELISA assay to endemic countries^92^. This test uses whole-virus antigen propagated in mouse brain, it takes over two days to perform, and the reagents exhibit lot-to-lot variation (not all reagents are supplied by US CDC); storage conditions may also influence the quality of results. Prior standardization is therefore required in each practicing locale, which restricts the test to well-trained laboratories. Despite these limitations, the availability of these reagents has for years enabled IgM testing by laboratories in endemic regions. The results for an improved MAC-ELISA kit from the US CDC employing antigen produced in Vero cells with lyophilized and stabilized reagents have been published recently^93^. The test can be run in one day and is intended for standard laboratories. Because it uses whole-virus antigen, however, it inherits the cross-reactivity risk of earlier protocols. Data on field applications of this assay are urgently needed as it is currently one of the few reliable options for YF serology in many laboratories. An IgM capture ELISA using new monoclonal antibodies against YFV has been described recently and has a promising sensitivity and specificity profile;^94^ however, these reagents are not yet widely available.

We know of four commercial tests currently available for IgM- or IgG-based serological diagnosis of YF. An immunofluorescence assay is available from EUROIMMUN AG (Lübeck, Germany) in 5- or 10-sample packaging. Each serum sample is reacted in parallel against an antigenic substrate (whole virus in infected cells) and a non-antigenic control (non-infected cells), which favors better interpretation of the results and facilitates the identification of false positives. The test was validated by the manufacturer on 300 European serum samples (150 from Swiss YFV-17D vaccinees and 150 from German blood donors) with an overall specificity of 96% in the IgM version of the assay and 94.7% for IgG and an overall sensitivity of 94.4% for IgM and 94.7% for IgG^95^. A YFV seropositivity of 4% for IgM and 6% for IgG was reported in the negative control group. This result exceeds the proportion of YFV vaccinees among the general German population, and results from these tests must therefore be interpreted with caution. Low sensitivity was observed toward IgM antibodies in the sera of YF-17D vaccinees^79^. Nevertheless, this commercial assay is the only one with available validation data. The manufacturer also sells multiplex assays in which sera are tested against several antigens in parallel; the assays including YFV are as follows: Flavivirus Mosaic 1 (TBEV, WNV, JEV, and YFV), Flavivirus Profile 2 and Flavivirus Mosaic 3 (TBEV, WNV, JEV, YFV, and DENV 1–4) and Arbovirus Profile 3 (ZIKV, CHIKV, DENV, TBEV, WNV, JEV, and YFV). Performance varies among the different antigens, along with the reported specificity profile. The proportion of positive sera for anti-DENV antibodies that presented an anti-YFV positive result is 100% with respect to IgG detection and 22.2% with respect to IgM detection. Likewise, anti-JEV positive sera are anti-YFV-positive in 100% (IgG) and 33.3% (IgM) of cases; and the figures for anti-WNV positive samples are 91.7% (IgG) and 33.3% (IgM). No data are available for cross reactions on Zika-positive samples. Other issues to consider in using IIF assays are the requirement for experienced technical personnel at the interpretation stage, the small number of samples that can be assayed in parallel, and the higher cost per test relative to other routine methods.

ELISA tests for IgM or IgG antibodies in 96-well plates (human yellow fever virus IgM/IgG ELISA kit) are available from Abbexa Ltd. (Cambridge, UK). No data on the performance of this assay have been reported by the manufacturer. MyBiosource, Inc., (San Diego, CA, USA) manufactures sandwich ELISA kits (Qualitative Human Yellow Fever Antibody IgM (YFV-IgM) or IgG (YFV-IgG)) in 48- and 96-sample formats and provides figures for intra- and inter-assay precision. No data are available on assay validation in regard to sensitivity, specificity or cross-reactivity. This assay is labeled for in vitro research only and not for diagnostic use. The Tariki YF-ELISA (Tariki Fiebre Amarilla IgM) is an IgM capture ELISA produced and sold in Peru since 2013 by the National Institute of Health of that country. The reported overall sensitivity is 95% (95% confidence interval: 87–100%) with 98% specificity (95% confidence interval: 87–100%)^96^. However, few data are available on the validation procedure for this test or its wider application in laboratories of the region.

### Other confirmatory assays for the diagnosis of YF

#### Histology and IHC

Even after the introduction of molecular methods, histological (hematoxylin-eosin staining) and immunohistochemical techniques continue to be valuable to reference laboratories as they provide supportive diagnoses in deceased cases and are useful for investigating epizootics. They provide a reliable diagnosis when antemortem serum or blood samples are not available, when specimens were not stored in conditions suitable for genome detection or viral isolation, or when hemolytic or autolytic processes are present.

The typical YF lesion is marked by lytic necrosis associated with hepatocyte apoptosis in the mid-zone of the liver lobule; cells bordering the central vein and portal triads are spared, and macro- and microvacuolar fatty changes can be observed in centrilobular cells. Eosinophilic degeneration of hepatocytes results in the formation of Councilman bodies and intranuclear eosinophilic granular inclusions. There is no disruption of the reticular architecture of the liver, and in nonfatal cases, healing is complete without postnecrotic fibrosis. Tubular necrosis in the kidneys is also observed frequently. The classical pathognomonic histological features of YFV infection are present only during the acute or late acute stages of the disease. Therefore, given a histological pattern of nonspecific hepatitis, ruling out a diagnosis of YF is contingent on additional evidence from serological and molecular tests.

The pathologic changes of YF-associated disease in NHPs are not fully resolved, and merit further study comparing the pathological findings in humans and other primates^97^. Divergences in the histopathological features of naturally and experimentally infected howler monkeys (*Alouatta*), where hepatic inflammatory mononuclear cell infiltration and hemorrhage are more pronounced than in humans or other primate species, must be carefully considered; otherwise, the diagnosis could be misleading as well as the identification of epizootics^98^.

Viral antigens can be detected in Kupffer cells and hepatocytes by IHC using YF-specific murine monoclonal antibodies or polyclonal rabbit sera. In addition, YFV antigens can also be detected in renal tubular epithelium and in groups of myocardial fibers^68^. YFV antigens and YFV RNA have been detected in the liver, kidney, spleen, lung, brain, and heart of deceased patients^56,68^, indicating that viral replication is not restricted to the liver and kidney, the major target organs.

Samples for IHC are preferably fixed using 10% neutral buffered formaldehyde^99^ and embedded in paraffin. Formaldehyde fixation prevents degradation and facilitates the manipulation and transport at room temperature of inactivated specimens to the reference laboratory; this effect is of great practical importance for field work and the investigation of epizootics in remote locations. The preferred protocol for YFV IHC uses specific antibodies against YF and the avidin-biotin complex technique. The quality, specificity, and careful validation of primary antibodies at laboratories are crucial for reliable results. The US CDC and the Evandro Chagas Institute (Brazil) produce and standardize qualified primary antibodies for this purpose. Different commercial reagents for detection are available, but the MACH-4 AP system (Biocare) is presently recommended as it provides increased sensitivity with minimal background by using a polymer-based detection system^100^.

Histopathological and IHC studies are laborious and require specific technical capability and expertise to provide reliable results; hence, they are practiced as reference techniques in expert laboratories.

#### Virus isolation

Laboratories embarking on YFV isolation must first establish appropriate biosafety practices (see Biosafety below). YFV can be isolated from blood collected during the initial febrile illness and from post-mortem tissues. The virus can be propagated in a variety of cell lines, including monkey epithelial and kidney fibroblasts (MA-104, Vero, LLC-MK2); rabbit- (MA-111) and baby hamster-derived lines (BHK); and *Ae. pseudoscutellaris* (AP-61) and *Ae. albopictus* (C6/36) mosquito cells. YFV may produce a cytopathic effect (CPE), and plaque formation is inconsistent and variable from strain to strain. While some strains produce detectable CPE or plaques within 1 or 2 days, many others require observation of the cells for 7–10 days. When CPE or plaques indicate that a virus has been isolated, the presence of viral RNA or antigens can be confirmed by RT-PCR or direct immunofluorescence using monoclonal antibodies.

YFV has been efficiently isolated by intrathoracic inoculation of mosquitoes and intracerebral inoculation of suckling mice or hamsters. Because of the requirement for laboratory animals and the availability of faster and simpler alternative protocols, this procedure is no longer recommended for routine diagnostic purposes. In addition, the efficiency of YFV isolation from clinical samples is greatly influenced by the presence of antibodies against the virus, sample storage conditions^55^, the isolation system implemented^101^, and the presence of metabolic products that can be detrimental to the growth of the virus on cell culture^68^.

## Biosafety

YFV is a risk Group 3 pathogen in the WHO and European classification and should be handled in a Biosafety Level 3 (BSL3) laboratory^102,103^. Depending on the epidemiological context of the country of origin of the samples (i.e., the presence of other hemorrhagic fever viruses that could be included as a differential diagnosis), laboratory facilities and procedures appropriate to Laboratory Containment—BSL3 or higher should be in place for viral isolation. Work should be carried out only by staff vaccinated against YFV at least 10 days prior to any handling of the virus or samples from suspected cases. Over 40 instances of professionally acquired YFV infections were reported in the pre-vaccine era. These cases included a physician caring for a patient, laboratory staff handling biological samples from infected patients or laboratory animals, and one case of transmission from the bite of an infected mosquito^38,104–107^.

Standard inactivation measures for risk group 3 pathogens are applicable. YFV is inactivated by 2% glutaraldehyde^108^, β-propiolactone, 2–3% hydrogen peroxide, 70% ethanol, 500–5000 ppm chlorine, 3–8% formaldehyde, 1% iodine and phenol iodophors, or 0.5% phenol with detergent^109^. Furthermore, YFV may be inactivated by heat at >50°C for 30 min and by gamma irradiation^109^.

## Concluding remarks

Astonishingly, for a well-known pathogen such as YFV, few diagnostic assays have been extensively validated using clinical samples from YF natural infections against different backgrounds of co-circulating flaviviruses. Performance statistics in terms of clinical-laboratory correlation are scarce for the available molecular methods. and only isolated cases have been reported, which have mainly occurred in travelers. Studies with sufficient statistical power are needed on the efficiency of YFV detection by molecular methods involving a follow-up of viraemia over time and examination of non-blood body fluids in parallel. Most reports on the persistence of viraemia have arisen from non-systematic observations in which YFV was generally identified by virus isolation, a technique with shortcomings in a diagnostic setting (as discussed above) and lower sensitivity than more recent molecular methods.

The analysis of body fluids other than serum or blood may widen the diagnostic window in natural infection cases, such as in ZIKV infection, where the pathogen has been detected in urine and semen. The possibility of detecting YFV in urine for longer periods than in serum^60,62^ warrants further investigation of urine as a useful diagnostic sample.

Detection of the NS1 antigen in the sera of acute YF cases holds promise for use as an alternative diagnostic target that affords high sensitivity and specificity in the early diagnosis of the disease^33^; however, available data evaluating this approach are currently limited to a recent publication^110^.

Current serological tests are unable to discriminate between cross-reactive flaviviral antibodies and between vaccine-acquired immunity and immunity from natural infection. The limited offering of commercial tests, scant data on their performance in the diagnosis of YF, and lack of well-defined validation panels hinder the rapid deployment of serological diagnostics during outbreaks.

Lastly, the implementation of YF diagnostic tools by regional laboratories in endemic countries remains challenging. Building up laboratory capacity and capability at the regional level would streamline case detection and foster the timely identification of new areas of transmission by removing bottlenecks at national reference laboratories that become overextended during epidemics and are forced to devote their resources to routine diagnosis. In this scenario, standardization of the assays used in reference laboratories (as currently recommended in the Americas), the establishment of regular quality control programs and interlaboratory comparisons using well-defined standards can provide insight into procedures or working protocols that need to be revised to improve detection capability and detect bias or uncertainties in test results related to the diagnostic laboratories. Furthermore, there is a strong need for standardization of the YFV case/laboratory definition across the Americas and Africa. A strong commitment would be required from authorities to invest heavily in laboratory equipment, logistics, staff training, quality assessment programs, and overall resource sustainability. Addressing the needs of remote laboratories in endemic regions entails developing affordable point-of-care YF diagnostics tests that must be easy to transport, run and interpret. Rigorous evaluation of new diagnostics tools before deployment will be essential. In addition, reference laboratories in non-endemic countries must be prepared and capabilities must be assessed to detect YF in returning travelers as an increasing number of cases have been exported related to the current outbreak in Brazil. For Europe, special attention is required in countries with endemic or intermittent presence of *Ae. aegypti* and *Ae. albopictus*.
